# Survival benefit associated with metformin use in inoperable non-small cell lung cancer patients with diabetes: A population-based retrospective cohort study

**DOI:** 10.1371/journal.pone.0191129

**Published:** 2018-01-12

**Authors:** Min-Chun Chuang, Yao-Hsu Yang, Ying-Huang Tsai, Meng-Jer Hsieh, Yu-Ching Lin, Chin-Kuo Lin, Pau-Chung Chen, Tsung-Ming Yang

**Affiliations:** 1 Department of Pulmonary and Critical Care Medicine, Chiayi Chang Gung Memorial Hospital, Chiayi, Taiwan; 2 Graduate Institute of Clinical Medical Sciences, College of Medicine, Chang Gung University, Taoyuan City, Taiwan; 3 Department of Traditional Chinese Medicine, Chiayi Chang Gung Memorial Hospital, Chiayi, Taiwan; 4 Health Information and Epidemiology Laboratory of Chang Gung Memorial Hospital, Chiayi, Taiwan; 5 School of Traditional Chinese Medicine, College of Medicine, Chang Gung University, Taoyuan, Taiwan; 6 Division of Pulmonary and Critical Care Medicine, Department of Internal Medicine, Chang Gung Memorial Hospital-Kaohsiung Medical Center, Kaohsiung, Taiwan; 7 Institute of Occupational Medicine and Industrial Hygiene, National Taiwan University College of Public Health, Taipei, Taiwan; 8 Department of Environmental and Occupational Medicine, National Taiwan University Hospital and National Taiwan University College of Medicine, Taipei, Taiwan; University of South Alabama Mitchell Cancer Institute, UNITED STATES

## Abstract

**Objective:**

To evaluate the effects of metformin use on the survival of inoperable non-small cell lung cancer (NSCLC) patients with diabetes using the Taiwanese National Health Insurance Research Database.

**Research design and methods:**

In total, 7,620 patients were eligible in this study, among them, 3,578 patients were metformin users and 4,042 were non-users. Propensity score matching was used to reduce possible confounding factors. In total, 4,182 patients (2,091 matched pairs) were included in the matched cohort. Cox proportional hazard model with time-dependent covariate were also applied to evaluate the association between metformin use and overall survival (OS).

**Results:**

A total of 3,578 patients were metformin users at the time of diagnosis of NSCLC. Cox proportional hazard model with time-dependent covariate revealed that metformin use was associated with a significantly longer OS (HR: 0.85, 95.0% CI: 0.80–0.90). The survival benefit of metformin use was maintained after propensity score matching at a ratio of 1:1 (HR: 0.90, 95.0% CI: 0.84–0.97).

**Conclusions:**

Metformin use is associated with longer OS in inoperable NSCLC patients with diabetes, suggesting a potential anti-tumorigenic effect for metformin. Further research is needed to investigate the actual role of metformin in the treatment of NSCLC patients with diabetes.

## Introduction

Despite remarkable improvements in diagnosis and treatment over the last few decades, lung cancer remains the leading cause of cancer-related mortality worldwide, with approximately 1.4 million deaths per year [1]. Lung cancer is responsible for approximately 27% of all cancer-related deaths in America [2] and approximately 20% of all cancer-related deaths in Taiwan [3]. About 80–85% of lung cancers are non-small cell lung cancer (NSCLC), and around 65% of NSCLC patients present with advanced stage IIIB or IV disease upon diagnosis [4].

Platinum-based chemotherapy remains one of the major treatment modalities in NSCLC for both patients without sensitive mutation for targeted therapy and those who acquired resistance to targeted therapy after treatment [5]. Despite the significant side effects and toxicities, however, chemotherapy provided limited benefit on overall survival (OS) in advanced stage NSCLC [6]. Therefore, there is an urgent need to discover and develop new strategies to improve the therapeutic efficacy of NSCLC.

Recently, a growing interest has emerged to identify the anti-cancer effect of drugs previously approved for non-cancerous indications. This approach, commonly known as drug repurposing or drug repositioning, entails finding novel therapeutic indications for an effective drug with limited side effects and reduced cost [7]. Recent studies have demonstrated promising anti-cancer activity for many of these medications in lung cancer [8]. Among these medications, metformin, an oral biguanide agent used for the treatment of type 2 diabetes, has gained much attention because of its potential anti-cancer effects *in vitro* and *in vivo* [9, 10]. Possible mechanisms for anti-cancer activity of metformin include inhibition of the unfolded protein response, induction of apoptosis, reductions in the levels of circulating insulin, and activation of specific metabolic pathways, such as liver kinase B1 and adenosine monophosphate-activated protein kinase (AMPK) [11, 12].

Meta-analyses of observational studies [13, 14] suggest that metformin use was associated with an overall reduction in cancer incidence in patients with diabetes. However, there are also studies showing that metformin use was not associated with reduced risk of cancer [15, 16]. In addition to possibly reducing the risk of cancer, metformin use has been associated with improved survival in diabetes patients with breast, prostate, and colorectal cancers [17–19]. Metformin use has also been found to improve chemotherapy outcomes and survival for NSCLC patients with diabetes [20]. Another population-based retrospective cohort study demonstrated a survival benefit of metformin use in stage IV NSCLC patients with diabetes [21]. However, the efficacy of metformin use on the outcomes of lung cancer patients has yet to be validated in different ethnicities and larger sample sizes.

In this study, we used the National Health Insurance Research Database (NHIRD) of Taiwan to investigate the effect of metformin use on the survival of inoperable NSCLC patients with diabetes.

## Materials and methods

### Ethics statement

The study was approved by the Institutional Review Board committee of Chiayi Chang Gung Memorial Hospital (Chiayi, Taiwan) (No. 104-8058B). As all personally identifiable information was encrypted prior to the release of the Taiwanese NHIRD, the need for informed consent was waived for this study.

### Data source

This retrospective longitudinal study was based on the NHIRD, which is released and managed by the Taiwan National Health Research Institute. The National Health Insurance Program is a mandatory social insurance program established by the Taiwanese government. It has provided comprehensive health care for all Taiwanese citizens since March 1, 1995 and currently covers approximately 99% of the national population. The NHIRD comprises the enrollment files, claims data, catastrophic illness files, and drug prescription registry. It represents one of the largest nationwide health care service databases in the world. The diagnostic accuracy of the Taiwanese NHIRD has been validated previously for major diseases [22].

The Registry for Catastrophic Illness Patient Database (RCIPD) is a subset of the NHIRD. It is a registry system for catastrophic illnesses, including cancer, end-stage renal disease, cirrhosis, congenital abnormalities, and autoimmune disease. The Bureau of National Health Insurance performs rigorous validations of the cancer diagnoses. At least two independent clinical physicians review the medical records and laboratory, histological, and imaging data of each patient who applies for catastrophic illness certification. Accordingly, the accuracy of cancer diagnosis in the RCIPD is expected to be reliable.

### Study cohort

A population-based retrospective cohort study was conducted using the NHIRD between January 1, 2000 and December 31, 2013. Lung cancer was defined using a compatible International Classification of Disease, Ninth Revision, Clinical Modification code (ICD-9-CM code 162) from the RCIPD (n = 104,963). The index date was defined as the date of first medical visit with an ICD-9 CM code for lung cancer. Lung patients with diabetes (n = 14,399) were identified from the lung cancer cohort using the following inclusion criteria: 1. Code for diabetes (ICD-9-CM code 250). 2. Pharmacy claims for ≥1 diabetic medicine for >28 defined daily doses in outpatient records before lung cancer was diagnosed. 3. Patients without coexisting malignancies other than lung cancer.

**Fig 1:**
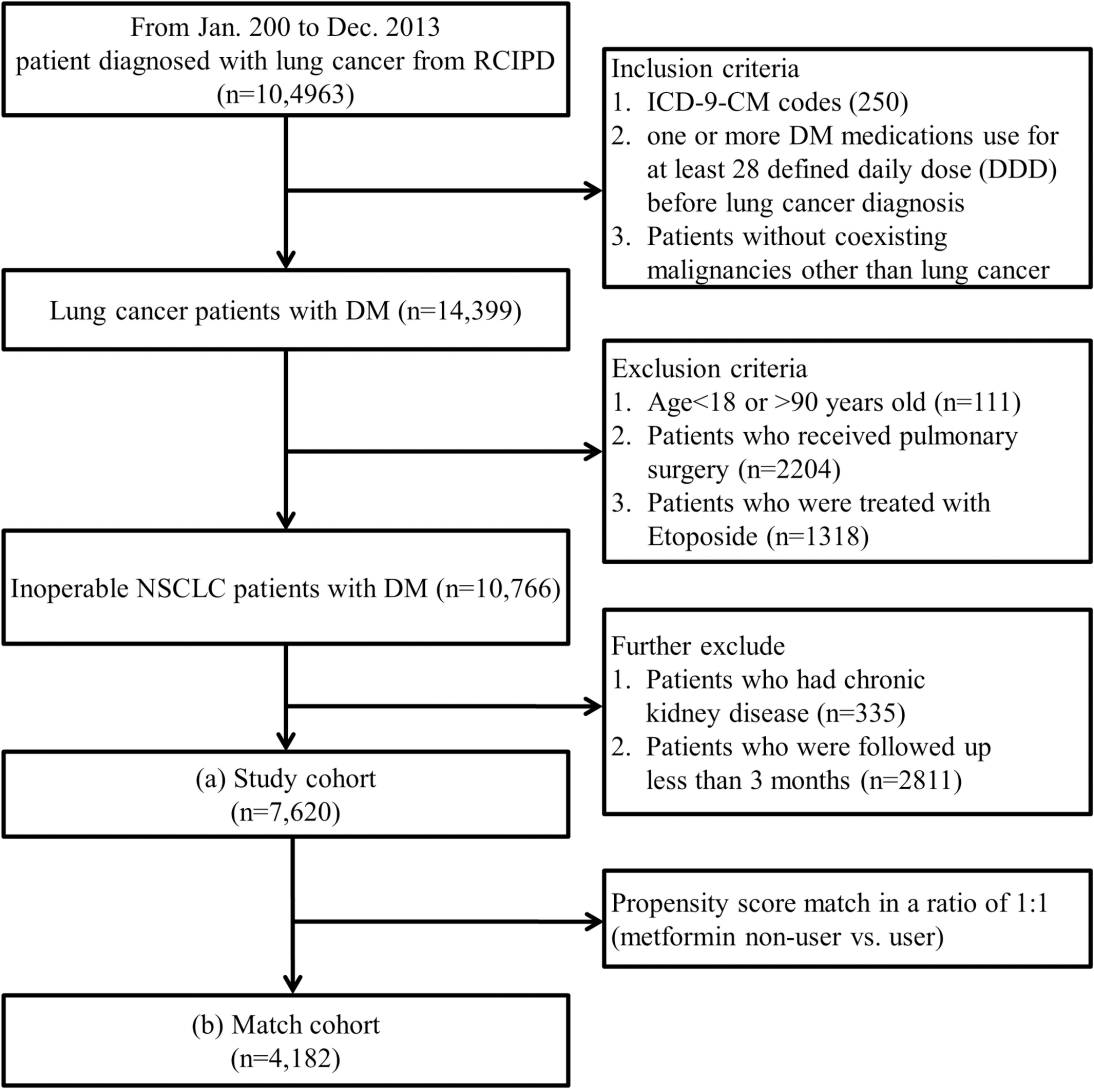
Flow chart of patient selection from the Registry for Catastrophic Illness Patient Database (RCIPD). ICD-9-CM, International Classification of Diseases, Ninth Revision, Clinical Modification; NSCLC, non-small cell lung cancer.

Patients <18 or >90 years of age (n = 111) were excluded. Although histological confirmation was required for issuing catastrophic illness certification of lung cancer, information on cell type and the clinical stage of the lung cancer was not available in the RCIPD. Patients treated with etoposide (n = 1,318) were suspected of having small cell lung cancer and were therefore excluded from this study. Operable lung cancer patients (n = 2,204) were defined as those having insurance claims for pulmonary surgery, including wedge resection, segmentectomy, lobectomy, and pneumonectomy, and were also excluded from this study. Metformin has been regarded as contraindicated in patients with chronic kidney disease. In addition, although cisplatin is widely accepted as the basic component of NSCLC chemotherapy, it is also contraindicated in patients with renal impairment. Patients with chronic kidney disease or end-stage renal disease (n = 335) were excluded because they may not receive metformin and standard cisplatin-base chemotherapy, and thus would negatively affect the analysis of the association between metformin treatment and NSCLC survival. Patients with a follow-up time of <3 months (n = 2,811) were also excluded, leaving 7,620 patients in the final analysis (Fig 1).

Each patient was followed up until 6 years after the index date, death, or the end of 2013. Death was defined as withdrawal of the patient from the NHI program. Metformin users are defined as those who use metformin for >28 defined daily doses after NSCLC diagnosis.

### Matched cohort

To confirm the association of metformin use and lung cancer survival, propensity score analysis was used to reduce possible confounding factors such as the Charlson Comorbidity Index (CCI), the adapted Diabetes Complication Severity Index (aDCSI), sociodemographic characteristics (age, sex, income, and level of urbanization), diabetes drugs other than metformin, and lung cancer treatments. The performance of the aDCSI in predicting the risk of hospitalization and health care cost has been validated previously [23] and can serve as an effective tool for stratifying diabetic patients for population-based studies. Metformin users and non-users were randomly frequency-matched according to propensity scores at a ratio of 1:1. In total, 4,182 insured adults (2,091 matched pairs) were included in the matched cohort.

### Primary study outcome

The primary study outcome was OS, determined from the NHIRD. Survival times were calculated as the time interval between the index date of lung cancer diagnosis and the date of death (defined as the date of withdrawal from the insurance system).

### Statistical analyses

Cox proportional hazard models with time-dependent covariate were used to compute hazard ratios (HRs) and the accompanying 95.0% CIs after adjustment for the CCI, the aDCSI, sociodemographic characteristics (age, sex, income, and level of urbanization), diabetes drugs other than metformin, and lung cancer treatments. The outcomes of different patient groups, stratified according to sex, age, the CCI, the aDCSI, diabetes drugs other than metformin, and lung cancer treatments, were also analyzed. All statistical analyses were conducted using SAS statistical software for Windows, version 9.4 (SAS Institute Inc., Cary, NC, USA). A two-tailed p < 0.05 was considered statistically significant.

## Results

In total, 3,578 patients were metformin users after NSCLC diagnosis. The median time of metformin use was 0.8 (interquartile range [IQR], 0.4–1.5) years, with a mean of 1.2 ± 1.2 years. Metformin users were younger (p < 0.001) and more likely to have fewer comorbidities (p < 0.001) than non-metformin users. Metformin users were also more likely to receive diabetes drugs other than metformin and lung cancer treatments compared to non-metformin-treated patients (p < 0.001). All covariates were well balanced after adjusting for propensity scores (Table 1). The metformin median dose was 140.56 DDD in study group and 115.75 DDD in matched group.

**Table 1:** Demographic and clinical characteristics of patients in the matched (n = 4,182) cohorts.

Characteristic	Matched Cohort		
	Metformin	No Metformin	p-value
	(n = 2,091)	(n = 2,091)	
Age, years (mean ± SD)	71.2 ± 9.4	71.7 ± 9.5	0.100
Sex, n (%)			0.431
M	1,261 (60.3)	1,236 (59.1)	
F	830 (39.7)	855 (40.9)	
Income (NTD), n (%)			0.996
0 (Dependent)	470 (22.5)	464 (22.2)	
1–15,840	372 (17.8)	373 (17.8)	
15,841–25,000	975 (46.6)	981 (46.9)	
≥25,000	274 (13.1)	273 (13.1)	
Urbanization, n (%)			0.741
1 (City)	545 (26.0)	567 (27.1)	
2	918 (43.9)	883 (42.2)	
3	382 (18.3)	390 (18.7)	
4 (Village)	246 (11.8)	251 (12.0)	
aDCSI, n (%)			0.780
0	1,535 (73.4)	1,527 (73.0)	
≥1	556 (26.6)	564 (27.0)	
CCI, n (%)			0.665
<6	1,072 (51.3)	1,086 (51.9)	
≥6	1,019 (48.7)	1,005 (48.1)	
Type 2 diabetes medication, n (%)			
Insulin	523 (25.0)	504 (24.1)	0.495
Sulfonylureas	1,446 (69.1)	1,439 (68.8)	0.815
Meglitinides	186 (8.9)	194 (9.3)	0.667
Thiazolidinediones	135 (6.5)	124 (5.9)	0.480
Alpha-glucosidase inhibitors	158 (7.6)	167 (8.0)	0.603
Dipeptidyl peptidase 4	125 (6.0)	130 (6.2)	0.747
Lung cancer treatment, n (%)			
Chemotherapy	1,193 (57.0)	1,207 (57.7)	0.662
CCRT	299 (14.30)	322 (15.40)	0.3172
Erlotinib	179 (8.6)	176 (8.4)	0.868
Gefitinib	268 (12.8)	269 (12.9)	0.963
Radiotherapy	737 (35.2)	763 (36.5)	0.402
None	606 (29.0)	584 (27.9)	0.455

aDCSI, adapted Diabetic Complication Severity Index; CCI, Charlson comorbidity index; F, female; M, male; NTD, New Taiwan dollar; SD, standard deviation; CCRT, Concurrent chemoradiotherapy

**Fig 2:**
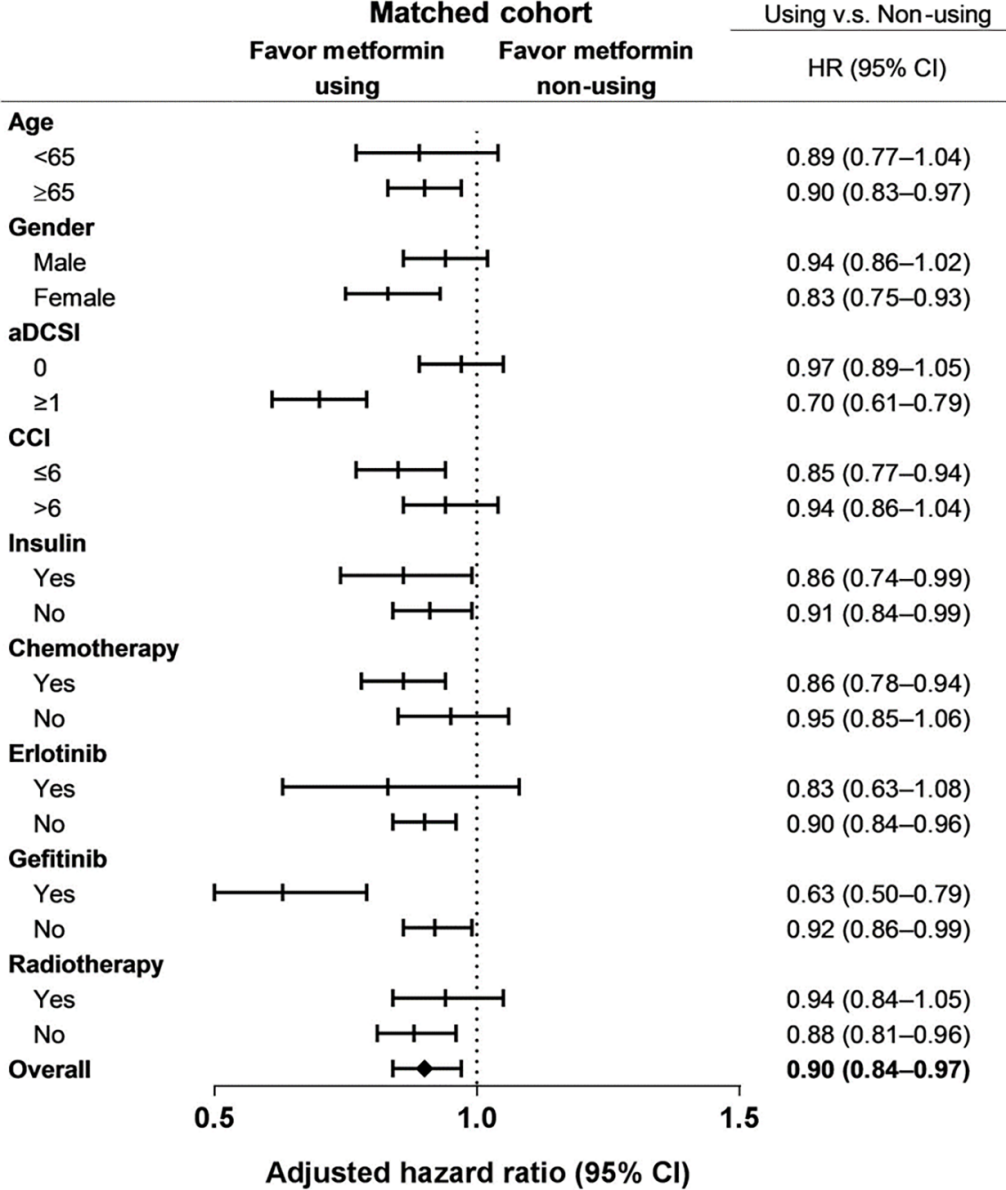
Subgroup analysis of adjusted hazard ratios (HRs) of risk factors for metformin-related mortality in the matched cohorts.

Cox proportional hazard model with time-dependent covariate showed that metformin use was associated with a significantly longer OS (HR: 0.85, 95.0% CI: 0.80–0.90). The survival benefit of metformin use was maintained after propensity score matching at a ratio of 1:1 (HR: 0.90, 95.0% CI: 0.84–0.97; Fig 2).

Subgroup analysis stratified by age, sex, the CCI, and the aDCSI also revealed a significant survival benefit in metformin users. The survival benefit of metformin use remained significant when the subgroup analysis was repeated by propensity score matching (Fig 2).

## Conclusion

In this population-based cohort study, we observed a significantly longer OS in patients treated with metformin in NSCLC patients with diabetes. This finding offered further evidence in support of the potential anti-tumorigenic effects of metformin in NSCLC patients.

Metformin, one of the most widely prescribed oral hypoglycemic agents, has received significant attention over the last decade due to its potential anti-tumorigenic effects. In 2005, Evans et al. [24] reported that among type 2 diabetes patients, those treated with metformin had a reduced risk of cancer compared to those who were untreated. In 2006, Bowker et al. [25] reported that type 2 diabetes patients exposed to metformin had a lower cancer-related mortality rate than those exposed to sulfonylureas or insulin. Furthermore, metformin use has been reported to improve survival outcomes in type 2 diabetes patients with several types of solid tumors [17–19]. Since 2011, a number of studies [21, 22, 26–34] have investigated the prognostic benefit of metformin use in NSCLC patients with diabetes. However, the findings in these studies were inconsistent, and more evidence will be needed to elucidate the actual role of metformin in NSCLC treatment. The present study showed that metformin use is associated with a longer OS in inoperable NSCLC patients with diabetes. Using a big data analysis approach, this study provided further evidence in support of the potential anti-tumorigenic effect of metformin in NSCLC.

The possible mechanism of the anti-cancer effect of metformin in NSCLC includes a direct inhibitory effect of metformin on tumor cell growth and the synergistic effects of metformin and NSCLC treatments. The direct anti-cancer effects of metformin in NSCLC have been demonstrated in previous studies [9, 35]. Although the precise mechanism(s) are not fully understood, the anti-tumorigenic effects may be attributed to the insulin-independent [36] and insulin-dependent actions of metformin [37].

Metformin has also been found to exhibit synergistic effects with chemotherapy and target therapy and has thus been studied as an adjuvant treatment for NSCLC [38, 39]. In support of this theory, we found that among patients treated with chemotherapy and gefitinib, those receiving metformin treatment had a longer OS. In a preclinical study, metformin sensitized NSCLC cells to ionizing radiation, leading to an increased response to radiotherapy [40]. In our study, among patients treated with radiotherapy, metformin treatment conferred a significant survival benefit. These findings suggest that adding metformin to chemotherapy, target therapy, or radiotherapy might prolong the OS NSCLC patients with diabetes.

There were several strengths in this study. First, the nationwide population-based setting of this study enabled us to enroll a large number of patients who were considered to represent NSCLC patients in real world. Second, the study cohort was selected from a computerized database comprising all Taiwanese NSCLC patients diagnosed between January 1, 2000 and December 31, 2013; thus, eliminating the potential for selection bias. Third, findings were validated using an alternative approach. After matching metformin users and non-users at a ratio of 1:1, according to factors such as the CCI, the aDCSI, sociodemographic characteristics (age, sex, income, and level of urbanization), diabetes drugs other than metformin, and lung cancer treatments, the findings were found to be comparable between the two approaches.

However, the findings in this study should also be interpreted in light of its limitations. First, this is a retrospective study based on diagnostic codes and prescription records; thus, there is heterogeneity that exits between metformin user group and non-metformin user group. However, to overcome this limitation, patients who were not eligible for metformin use were excluded. In addition, propensity scores were used to adjust for potential confounders and showed that the survival benefit was maintained in the metformin user group. Second, although NSCLC patients identified from catastrophic illness certificates in the NHIRD are valid and highly reliable owing to the strict review system, information regarding cell type and the clinical stage of the lung cancer was not available in this database. In addition, several unmeasured confounders, including body mass index, smoking habit, and alcohol consumption, which are all associated with survival, were not included in the NHIRD. For this reason, this study was conducted in a cohort of inoperable NSCLC patients who were regarded as having advanced-stage disease. We believe that the mortality in this population was mostly cancer-related; thus, the potential effect of confounders of patient survival would be reduced. Third, it is difficult to confirm the exact dosage of drugs that were administered. Furthermore, it was assumed that all prescribed medications were taken by the patients. However, this could have resulted in an overestimation of the actual dosage due to a lack of adherence that inevitably exists. Forth, we defined metformin users as those who use metformin for >28 defined daily doses after NSCLC diagnosis. Immortal time bias may play a role and the protective effects of metformin may be an artifact of immortal time bias. We use time-dependent Cox regression for further analysis (yearly metformin use <28 DDD as reference group) to reduce the effect of immortal time bias, and the results still showed survival benefit in metformin users. Fifth, patient treated with curative or palliative intent were not addressed in this study. Surgical resection in the main modality for NSCLC treatment with curative intent. For early stage NSCLC patients who did not received surgical resection, curative radiotherapy is the major alternative. However, the information to differentiate whether a radiotherapy was curative or palliative intent was not available in the NHIRD. This limitation prohibits the NHIRD study to differentiate patients treated with curative or palliative intent.

The findings of this study suggest that among inoperable NSCLC patients with diabetes, metformin use is associated with an improved OS. Therefore, metformin should be considered as the treatment option of choice for inoperable NSCLC patients with diabetes. However, future prospective randomized clinical trials are needed to validate the anti-tumorigenic activities of metformin.
